# Impact of the COVID-19 Pandemic on parathyroid adenoma surgery: a single-center comparative experience

**DOI:** 10.1186/s12893-026-03624-6

**Published:** 2026-02-26

**Authors:** Bahadır Öndeş, Osman Gökhan Gökdere

**Affiliations:** Department of General Surgery, Malatya Turgut Özal University, Malatya, Türkiye Turkey

**Keywords:** COVID-19, primary hyperparathyroidism, parathyroid adenoma, parathyroidectomy, surgical outcomes

## Abstract

**Objective:**

The COVID-19 pandemic profoundly disrupted healthcare delivery worldwide, leading to delays and uncertainties in the management of various endocrine disorders, including primary hyperparathyroidism. This study aimed to assess the impact of the pandemic on surgical outcomes by comparing the clinical, biochemical, and perioperative results of patients undergoing parathyroidectomy during and after the pandemic.

**Methods:**

A retrospective review was conducted of patients who underwent surgery for primary hyperparathyroidism between March 2020–June 2021 (COVID period) and July 2022–December 2023 (post-COVID period). Demographic characteristics, clinical presentation, biochemical markers, radiological findings, operative time, length of hospital stay, and postoperative complications were analyzed.

**Results:**

Operative times were significantly longer during the COVID period, whereas hospital stays were shorter. While overall biochemical control was achieved in the majority of patients, persistent postoperative hyperparathyroidism was significantly more frequent during the pandemic period.

**Conclusion:**

Although the COVID-19 pandemic was associated with delayed presentation and increased perioperative challenges, parathyroidectomy remained an effective treatment option. However, higher rates of hypercalcemic crisis and persistent postoperative hyperparathyroidism highlight the clinical consequences of delayed surgical care during public health crises.

## Introduction

 Primary hyperparathyroidism (PHPT) is an endocrine disorder characterized by persistent elevation of parathyroid hormone (PTH) and serum calcium levels, and it is frequently asymptomatic at the time of diagnosis. The prevalence of PHPT in the adult population is estimated to be approximately 1% [1]. The most common cause of PHPT is parathyroid adenoma, which typically arises from a single parathyroid gland [2]. If left untreated, PHPT can lead to osteoporosis, nephrolithiasis, neuropsychiatric manifestations, and cardiovascular complications [3]. Therefore, once the diagnosis is established, surgical treatment is considered the gold standard for eligible patients [4].

The COVID-19 pandemic significantly affected healthcare delivery worldwide. Elective surgical procedures were particularly impacted, as healthcare resources were redirected primarily toward the management of COVID-19 patients, and many healthcare professionals were reassigned to COVID-specific units [5]. Within endocrine surgery, patients with primary hyperparathyroidism (PHPT) were often deprioritized, as urgent surgical indications are uncommon in the majority of cases [6]. Consequently, parathyroidectomies were frequently postponed during this period. However, the impact of these delays on patients’ clinical presentation and surgical outcomes has not yet been clearly elucidated in the literature [7].

In this study, we compared the clinical, biochemical, and surgical characteristics of patients who underwent parathyroidectomy for primary hyperparathyroidism (PHPT) during and after the COVID-19 pandemic. The objective was to elucidate the impact of pandemic-related conditions on patient characteristics, diagnostic pathways, and surgical outcomes, and to derive clinical insights that may inform the management of similar scenarios in potential future pandemics.

## Materials and methods

### Study Design and Patient Selection

This was a single-center, retrospective cohort study including patients who underwent parathyroidectomy for primary hyperparathyroidism (PHPT) at our institution. Patients were categorized into two groups according to the time of surgery: the COVID period (March 2020–June 2021) and the post-COVID period (July 2022–December 2023). All consecutive patients who underwent surgery for primary hyperparathyroidism and received a final histopathological diagnosis of parathyroid adenoma or hyperplasia were included.

Demographic data (age, sex), preoperative biochemical parameters, neck ultrasonography (USG) findings, and Tc-99 m sestamibi scintigraphy results were collected. Postoperative pathology reports and follow-up parathyroid function status (hypoparathyroidism or persistent/recurrent hyperparathyroidism) were also recorded. Patients with incomplete medical records, prior parathyroid surgery, or parathyroid carcinoma were excluded.

Patients with a histopathological diagnosis of parathyroid neoplasia or carcinoma were excluded from the final analysis.

### Definition of Postoperative Hyperparathyroidism

Postoperative hyperparathyroidism was defined as persistent hyperparathyroidism, characterized by elevated serum parathyroid hormone (PTH) levels with or without hypercalcemia within six months following surgery. Patients who achieved initial biochemical normalization but developed recurrent disease during long-term follow-up were not classified as having postoperative hyperparathyroidism.

### Surgical Technique and Intraoperative Management

All patients underwent parathyroidectomy performed by the same experienced endocrine surgery team. The surgical approach was determined based on preoperative localization studies, including neck ultrasonography and Tc-99 m sestamibi scintigraphy. A focused or unilateral neck exploration was preferred in patients with concordant imaging findings, whereas bilateral neck exploration was reserved for selected cases with non-localizing or discordant imaging.

Intraoperative parathyroid hormone (ioPTH) monitoring was routinely used to confirm biochemical success, defined as a decrease of more than 50% from baseline PTH levels within 10 min after gland excision. The operative strategy and use of intraoperative adjuncts did not differ between the COVID and post-COVID periods.

Concomitant thyroid procedures were performed only when clinically indicated based on preoperative evaluation and intraoperative findings.

### Statistical analysis

All data were analyzed using the Statistical Package for the Social Sciences (SPSS) software for Windows, version 22.0 (IBM Corp., Armonk, NY, USA). Continuous variables were tested for normality using the Kolmogorov–Smirnov test, along with skewness and kurtosis values. Normally distributed data were expressed as mean ± standard deviation (SD) and compared using Student’s *t*-test. Non-normally distributed variables were presented as median and interquartile range (IQR) and compared using the Mann–Whitney U test. Paired comparisons were assessed with the Wilcoxon signed-rank test. Categorical variables were expressed as numbers and percentages, and associations were analyzed using the chi-square test or Fisher’s exact test when assumptions for chi-square were not met. A *p*-value < 0.05 was considered statistically significant.

## Results

A comparison of the COVID and post-COVID groups revealed no statistically significant differences in gender distribution, parathyroid lesion localization on ultrasonography, scintigraphy utilization, pathological diagnosis, or incidence of postoperative hypoparathyroidism (*p* > 0.05 for all).

Notably, postoperative hyperparathyroidism was significantly more frequent in the COVID period compared with the post-COVID period (14.29% vs. 1.47%; χ²=7.62, *p* = 0.01), indicating a higher rate of persistent biochemical activity following surgery during the pandemic period (Table 1).

**Table 1:** Comparison of imaging findings, pathological characteristics, and postoperative outcomes between the COVID and post-COVID periods

Variable	Category	Group				X^2^	*p*-value
		COVID period (*n* = 63)		Post-COVID period (*n* = 68)			
		*n*	*%﻿*	*n*	%		
Gender	Male	13	20.63	19	27.94	0.95	0.33
	Female	50	79.37	49	72.06		
Ultrasonography	Performed	63	100.00	68	100.00		
Parathyroid lesion localization (USG)	Right	43	68.25	40	58.82	1.25	0.26
	Left	20	31.75	28	41.18		
Scintigraphy	Performed	51	80.95	53	77.94	0.18	0.67
	Not performed	12	19.05	15	22.06		
Pathology	Adenoma	58	92.06	64	94.12	1.61	0.45
	Hyperplasia	5	7.94	3	4.41		
Postoperative hypoparathyroidism	Present	4	6.35	1	1.47	2.12	0.15
	Absent	59	93.65	67	98.53		
Postoperative hyperparathyroidism	Present	9	14.29	1	1.47	7.62	0.01
	Absent	54	85.71	67	98.53		
Hypercalcemic crisis at presentation	Present	14	22.22	1	1.47	12.84	< 0.01
	Absent	49	77.78	67	98.53		

*χ²: Chi-square test; p<0.05 considered statistically significant

Regarding clinical presentation, hypercalcemic crisis at admission was significantly more frequent in the COVID period compared with the post-COVID period (22.22% [*n* = 14] vs. 1.47% [*n* = 1], χ²=12.84, *p* < 0.01).

Comparison of the COVID and post-COVID groups revealed no statistically significant differences in preoperative calcium, phosphorus, parathyroid hormone (PTH) levels, patient age, or tumor size (*p* > 0.05 for all). Similarly, postoperative biochemical parameters did not differ significantly between the two groups.

Significant differences reflecting increased operative complexity during the pandemic were observed in hospital length of stay, operative time, and lesion size measured by ultrasonography. The mean hospital stay was 1.83 ± 1.69 days (median: 2.0; range: 1–6) in the COVID group and 2.07 ± 2.20 days (median: 1.0; range: 1–14) in the post-COVID group (z = − 3.05; *p* < 0.01). The mean operative time was significantly longer in the COVID group (133.33 ± 49.54 min; median: 120.0; range: 60–300) compared with the post-COVID group (109.12 ± 36.62 min; median: 120.0; range: 60–180) (z = − 2.86; *p* < 0.01).

Moreover, ultrasonographically measured lesion size was significantly greater in the COVID group compared with the post-COVID group (13.86 ± 5.07 mm vs. 11.18 ± 4.22 mm, respectively; z = − 3.20; *p* < 0.01) (Table 2).

**Table 2:** Comparison of preoperative and postoperative biochemical parameters, demographic features, and perioperative outcomes between the COVID and post-COVID groups

Variable	COVID period (*n* = 63)		Post-COVID period (*n* = 68)		Between-group comparison
	X̅±SD	Median (Min-Max)	X̅±SD	Median (Min-Max)	
Preoperative calcium (mg/dL)	11.39 ± 1.17	11.3(8.0-16.5)	11.61 ± 1.27	11.6(8.3–15.1)	z:-1.70;p:0.09
Postoperative calcium (mg/dL)	8.66 ± 1.05	8.8(5.5–11.8)	8.89 ± 0.78	9.0(6.5–10.4)	z:-1.04;p:0.30
Preoperative PTH (pg/mL)	416.57 ± 723.80	188.0(65.0-3698.0)	317.74 ± 532.18	173.0(1.9–3064.0)	z:-0.69;p:0.49
Postoperative PTH (pg/mL)	28.97 ± 19.27	26.0(1.0-104.0)	39.37 ± 35.50	26.5(5.0-200.0)	z:-1.13;p:0.26
Preoperative phosphorus (mg/dL)	2.98 ± 1.11	2.7(1.7–6.9)	2.78 ± 0.93	2.6(1.3–7.4)	z:-0.57;p:0.57
Postoperative phosphorus (mg/dL)	3.22 ± 0.81	3.2(1.2–5.9)	3.44 ± 0.73	3.4(2.2–5.2)	z:-1.74;p:0.08
Tumor size (mm, pathology)	14.49 ± 5.40	14.0(5.0–27.0)	12.90 ± 5.00	12.0(5.0–30.0)	z:-1.71;p:0.09
Hospital stay (days)	1.83 ± 1.69	2.0(1.0–6.0)	2.07 ± 2.20	1.0(1.0–14.0)	z:-3.05; *p* < 0.01
Operative time (min)	133.33 ± 49.54	120.0(60.0-300.0)	109.12 ± 36.62	120.0(60.0-180.0)	z:-2.86;*p* < 0.01
Age (years)	54.35 ± 13.75	54.0(22.0–79.0)	53.57 ± 12.95	52.5(19.0–80.0)	z:-0.31;p:0.76
Lesion size (mm, USG)	13.86 ± 5.07	13.0(5.0–28.0)	11.18 ± 4.22	10.0(4.0–30.0)	z:-3.20; *p* < 0.01

*Wilcoxon test

**Mann-Whitney U test

## Discussion

The limited capacity of healthcare systems during the COVID-19 pandemic led to substantial delays in elective surgical procedures. In conditions such as primary hyperparathyroidism (PHPT), which are often asymptomatic but may cause long-term complications, this was associated with delayed presentation and treatment. In our study, the rate of hypercalcemic crisis was 22.2% in patients operated on during the pandemic, compared with 1.5% in the post-pandemic period (*p* < 0.01). This marked difference may reflect challenges in accessing healthcare services and delays in surgical management during the pandemic. Consistent with these observations, Tunca et al. reported a 54.5% reduction in parathyroid surgery volume in 2020 versus 2019 at a tertiary endocrine surgery center [8]. Taken together, these findings underscore the potential clinical importance of timely surgical intervention, even under extraordinary healthcare constraints.

One of the most clinically relevant findings of this study was the higher rate of postoperative hyperparathyroidism observed during the COVID-19 period. In the present series, postoperative hyperparathyroidism was defined as persistent disease rather than recurrence, reflecting incomplete early biochemical resolution after surgery. This finding may be associated with several pandemic-related factors, including delayed surgical referral, prolonged disease duration, and more advanced gland pathology at presentation. In addition, logistical constraints during the pandemic may have limited perioperative optimization and follow-up, potentially contributing to persistent biochemical activity despite technically successful surgery.

Nevertheless, when analyzing overall biochemical outcomes—specifically the reduction in serum calcium and PTH levels—both cohorts demonstrated meaningful improvement. This apparent discrepancy likely reflects the definition used for persistent disease, which emphasizes early biochemical patterns rather than long-term recurrence or clinical failure. In this context, the higher rate of persistent disease may be interpreted as a marker of early postoperative biochemical incompleteness rather than diminished surgical efficacy.

These findings suggest that, even in high-volume referral centers, public health crises such as the COVID-19 pandemic may influence the rate of complete early biochemical response in the management of benign parathyroid disease through delays in care. Nevertheless, parathyroidectomy appeared to maintain its surgical effectiveness, with both cohorts achieving substantial reductions in serum calcium and PTH levels and cure rates exceeding 95%. This indicates that, despite logistical challenges, overall surgical success was largely preserved [9].

Although surgical technique was not a comparative variable in this study, previously published data suggest that earlier referral and optimized surgical planning may contribute to improved postoperative recovery. Specifically, multidisciplinary analyses have demonstrated that minimally invasive and focused parathyroid approaches, combined with intraoperative parathyroid hormone monitoring (ioPTH), reduce postoperative hypocalcemia and enhance cure rates [10]. In randomized trials comparing unilateral (focused) and bilateral neck explorations, focused exploration yielded comparable long-term outcomes with fewer early postoperative morbidities. Moreover, timely surgical referral—facilitated by earlier clinical presentation in the post-pandemic cohort—may have enabled a more stable preoperative status, thereby potentially optimizing perioperative biochemical recovery and minimizing complications [11].

Evaluation of operative times revealed that surgeries conducted during the COVID-19 period were significantly prolonged. This extension cannot be solely attributed to patients presenting with more complex clinical states; pandemic-specific logistical constraints may also have contributed. Factors such as additional preparation required for donning personal protective equipment (PPE), extended operating room sterilization protocols, and occasional limitations in staff or equipment availability may all have played a role in lengthened operative durations. Notably, an international survey of surgeons reported that PPE use impaired visual communication, decision-making, and overall non-technical performance—factors that can increase operative times [12]. Furthermore, process-based analyses from the University Hospital of Ulm documented sustained delays in perioperative workflows attributable to COVID-19 restrictions, reflecting a broader system-level impact on operative efficiency [13].

A statistically significant difference was observed between the groups in terms of length of hospital stay, with early discharge during the pandemic being adopted as a strategy to minimize infection risk. The incidence and predictors of post-parathyroidectomy hypocalcemia have been explored in recent cohort studies, emphasizing the role of preoperative biochemical markers in determining postoperative outcomes [14]. Specifically, parathyroidectomy is associated with transient hypocalcemia in 15–30% of cases, whereas permanent hypocalcemia remains rare [15]. Importantly, no cases of permanent hypoparathyroidism, recurrence, or nerve injury were observed in either group. These findings are consistent with the high success rates and favorable safety profile of parathyroid surgery reported in the literature [16].

One of the major strengths of our study is that all procedures were performed by the same surgical team, thereby reducing operator-dependent variability. In addition, the relatively large patient cohort and the availability of detailed biochemical follow-up data enhanced the statistical robustness of our findings. Nevertheless, certain limitations should be acknowledged. The retrospective design, single-center setting, and lack of long-term follow-up data may limit the generalizability of our results.

## Conclusion

The COVID-19 pandemic had a measurable impact on the clinical presentation and perioperative characteristics of patients undergoing surgery for primary hyperparathyroidism. Patients operated on during the pandemic period more frequently presented with hypercalcemic crisis, larger parathyroid lesions, prolonged operative times, and a higher rate of persistent postoperative hyperparathyroidism, reflecting the consequences of delayed surgical referral and restricted access to elective care.

Despite these challenges, parathyroidectomy remained an effective treatment option, with satisfactory early biochemical outcomes in both periods. These findings underscore the importance of timely surgical management of benign parathyroid disease, even during public health crises, to prevent disease progression and optimize postoperative outcomes.

## Data Availability

The patient data and statistical analyses used to support the findings of this study are available in written form and can be provided by the corresponding author upon reasonable request.
